# Impact of Metal Source Structure on the Electrocatalytic Properties of Polyacrylonitrile-Derived Co-N-Doped Oxygen Reduction Reaction Catalysts

**DOI:** 10.3390/nano14231924

**Published:** 2024-11-29

**Authors:** Arseniy Kalnin, Ksenia Kharisova, Daniil Lukyanov, Sofia Filippova, Ruopeng Li, Peixia Yang, Oleg Levin, Elena Alekseeva

**Affiliations:** 1 Electrochemistry Department, St. Petersburg State University, 7/9 Universitetskaya nab., 199034 St. Petersburg, Russia; 2 State Key Laboratory of Urban Water Resource and Environment, School of Chemistry and Chemical Engineering, Harbin Institute of Technology, Harbin 150001, China

**Keywords:** oxygen reduction reaction, cobalt-doped carbons, catalytic performance, nitrogen-rich environment, methanol tolerance

## Abstract

The oxygen reduction reaction (ORR) plays a central role in energy conversion and storage technologies. A promising alternative to precious metal catalysts are non-precious metal doped carbons. Considerable efforts have been devoted to cobalt-doped carbonized polyacrylonitrile catalysts, but the optimization of their catalytic performance remains a key challenge. We have proposed a multifunctional active metal source strategy based on the cobalt complex with the ligand containing pyridine and azo-fragments. This complex simultaneously provides the nitrogenous environment for the Co atoms and acts as a blowing agent due to N_2_ extrusion, thus increasing the surface area and porosity of the material. This strategy provided the catalysts with a high surface area and pore volume, combined with the greater fraction of Co-N clusters, and a lesser amount and smaller size of Co metal particles compared to conventionally prepared catalysts, resulting in improved catalytic performance. In addition to strict 4-electron ORR kinetics and 383 mV overpotential, the novel catalysts exhibit limiting current values close to the Pt/C benchmark and greatly overcome the Pt in methanol tolerance. These results demonstrate the critical role of metal source structure and carbonization parameters in tailoring the structural and electrochemical properties of the catalysts.

## 1. Introduction

The electrocatalytic oxygen reduction reaction (ORR) plays a key role in the operation of fuel cells and metal-air batteries, and thus attracts considerable attention [1,2,3].

Carbon electrodes with platinum catalysts are commonly used in commercial alkaline fuel cells and metal-air batteries [4]. Pt-based catalysts are highly active in ORR [5,6]. However, high cost, poor stability, and low methanol tolerance of Pt have seriously hindered the commercial application of such catalysts [7,8,9].

Platinum metal group (PMG) free ORR catalysts based on the carbon-supported first-row transition metals, such as cobalt or iron, are approaching the Pt-based catalysts in terms of catalytic activity. Although many non-platinum catalysts are very efficient in the ORR, only the platinum catalyst is commercially available for the reaction. This is probably due to the fact that the production of non-platinum ORR catalysts requires complex methods and precursors that cannot be scaled up [10,11,12,13,14].

The efficiency of PMG-free ORR catalysts has been found to increase when the metal phase consists of a few atom clusters or single atoms on the surface of the carbon matrix [15]. However, the controllable synthesis of PMG-free ORR catalysts with a highly dispersed metal phase and developed surface is a challenge due to the agglomeration of metal clusters and carbon particles. In addition, a highly developed surface and porous structure is required for efficient mass transfer. Amorphous nitrogen-doped carbons (CN_x_) [16] also show great promise as a matrix for the PMG-free ORR catalysts, since the nitrogen sites on their surface stabilize the highly dispersed few-atom metal active centers in the catalyst, and provide additional sites for the coordination and reduction of the dioxygen, whereas the amorphous structure allows efficient mass transfer [17]. Thus, the nitrogen-doped carbon matrix acts as catalytic N sites while stabilizing some atom Co clusters on the main active moieties in catalysts (i.e., the M-N_x_ sites, where the transition metal as the center atom coordinates with N atoms) [18], increasing the ORR catalytic activity.

Binary composite systems consisting of carbon nanotubes coated with polymer complexes and doped with metal oxides offer a particularly effective solution for creating high-performance ORR catalysts. The synthesis of highly doped carbon materials for industrial and laboratory purposes typically involves the carbonization of organic precursors [19,20,21]. This procedure allows the composition and morphology of the carbon materials to be controlled by tuning the molecular and supramolecular structures of the polymers. To ensure the uniformity of material composition and structure, a precursor with a rigid structure is required to maintain the initial distribution of doping atoms during carbonization at elevated temperatures [22].

One of the preparation methods of a metal-containing catalyst is the introduction of metal into the structure of the polymer precursor. Another method, i.e., the impregnation of polymer precursors with solutions of metal salts, such as acetate, nitrate, etc., is considered to be more technologically efficient. To develop a commercially affordable and performance non-platinum catalyst, it is necessary to use widely accessible and inexpensive sources of carbon materials and the method of metal loading.

Among possible organic precursors for the metal, N-codoped carbons (M-CN_x_), and polyacrylonitrile (PAN) in particular, provide a more controllable carbonization process due to the “pre-cyclization” feature that forms the polycyclic N-heterocyclic ribbon at the initial stages of heating [22,23], which gives coordination sites for metal ions and affords high mass retention after carbonization. The resulting CN_x_ contains a large proportion of “pyridinic” nitrogen atoms, which are believed to ensure strong bonding with metal clusters and contribute to the ORR activity [24,25].

Furthermore, the structural features and nitrogen doping contribute to the bifunctional capabilities of these catalysts, enabling activity for both the oxygen reduction reaction (ORR) and the oxygen evolution reaction (OER), making them promising candidates for diverse electrochemical applications [16]. ORR catalyst efficiency depends on both morphology and composition.

The morphology of catalysts can be improved in several ways, e.g., by introducing pore formers, which release gaseous decomposition products strongly upon heating. In addition, various sophisticated nanostructured catalysts have shown good results. To illustrate, pyridinic N-doped porous carbon catalysts with a single Co atom were prepared by Ha et al. [26] using a lysozyme (Lys)-assisted ZIF-8. Pyridinic N could reduce electron localization around the Co centers, thereby lowering the energy barrier and facilitating the 4e^-^ ORR process. Gao et al. [27] devised a novel ZIF using an acetic acid (OAc)-assisted strategy. The Co ion exhibited two forms of coordination: the first was the established Co-imidazole coordination, and the second was a bridge Co ion between the OAc and the Zn node. OAc helped create high-density, accessible Co active sites by isolating Co ions and forming macropores during carbonization.

However, despite the excellent kinetic properties of the presented Co ORR catalysts [26,28,29,30,31], the complexity of their preparation procedures hinders their practical implementation. The same is true for bimetallic catalysts [28,32], which require researchers to vary metal ratios and, as a result, making it more of a challenge to control the morphology and size of the metal particles. Commercializing the PMG-free ORR catalyst therefore requires a simplified synthesis using affordable precursors. Still, the use of conventional metallic cobalt precursors, such as acetates and nitrates [30], increases the slope of the Tafel plot [28,30,32], making it necessary to develop a cobalt precursor that can produce Co-CN_x_ catalysts with better catalytic properties and morphology.

**Figure 1:**
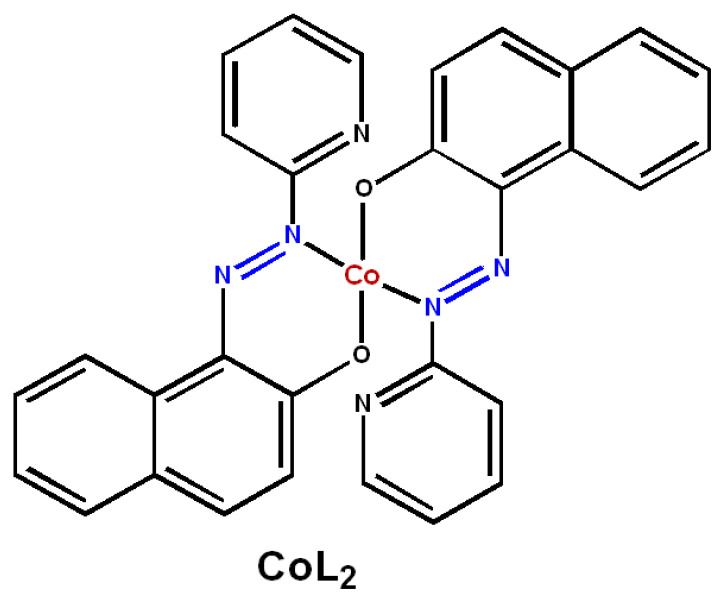
Structure of the CoL_2_ complex.

This work is focused on the development of the simplified and affordable synthesis of Co, N-codoped carbon (Co-CN_x_) ORR catalyst using a bifunctional metal precursor–cobalt complex with azo-containing ligand 1-(2-pyridylazo)-2-naphthol (CoL_2_, Figure 1) as a metal source.

One notable advantage of the catalyst obtained in this study is that the PAN as CN_x_ and 1-(2-pyridylazo)-2-naphthol used are common and commercially available compounds [33,34], which allow a number of stages of final catalyst synthesis to be reduced. Undoubtedly, the utilisation of cobalt has the potential to decrease production costs, which in the case of fuel cells is reported to be more than 50%, depending on the price of noble metal precursor (e.g., Pt/C) [35]. Also, CoL_2_ shows excellent solubility in the PAN due to a bulky organic periphery. At the same time, the mechanisms of N_2_ formation during the decomposition of azo dyes are well known [36] and, upon heating, the proposed ligand decomposes with the release of nitrogen [22,37]. Thanks to this, CoL_2_ during pyrolysis will act not only as a cobalt source but also as a pore-former, uniformly distributed within the bulk of the PAN. We assume that the combination of these factors ensures a homogeneous distribution of metal atoms throughout the catalyst, reduces the formation of a pure metal phase, and provides materials with a high surface area and a developed porous structure [22,36,37].

In this study, we will investigate the hypothesis that this nitrogen release contributes to the catalyst’s functional properties. The catalyst obtained from CoL_2_ will be compared with the one obtained from a conventional metal source, Co(OAc)_2_, using several physico-chemical methods. The ORR catalytic activity of these materials will be studied and compared in an alkaline medium using a rotating disk electrode (RDE) method.

## 2. Materials and Methods

Commercially available typical PAN with the average molecular weight of M_w_ = 150 kDa, Co(OAc)_2_-4H_2_O, and 1-(2-pyridylazo)-2-naphthol were purchased from Sigma Aldrich. A cobalt complex of 1-(2-pyridylazo)-2-naphthol), CoL_2_, was prepared as described in the literature [38].

**Figure 2:**
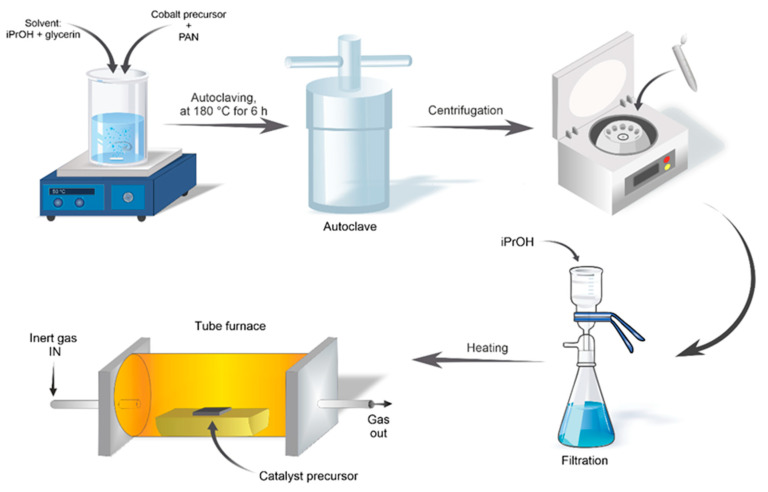
The scheme of the catalyst preparation.

To prepare the CoCN_x_ catalysts (Figure 2), a solution of 2 mg of PAN in 2.2 mL of N,N-dimethylformamide (DMF) was mixed with 0.35 mmol of corresponding cobalt precursor. The resulting solution was then poured into a mixture of 20 mL of isopropanol (*i*-PrOH) and 2 mL of glycerol, and the resulting mixture was pre-heated to 50 °C and homogenized for 20 min. The obtained dispersion was sealed in a 150-mL stainless steel pressure reactor equipped with a polytetrafluoroethylene (PTFE) gasket and heated to 180 °C for 6 h. After heating, the precipitate was collected by centrifugation, washed thoroughly with *i*-PrOH, and dried at 80 °C for 6 h and then for 12 h under vacuum at 80 °C. Then the pretreated sample was heated in a tube furnace equipped with a quartz tube at a specified temperature for 2 h. The heating process was conducted in the flow of 99.995% purity argon gas, and after completion the sample was cooled down under argon and ground manually using a mortar.

Surface morphology and particle size analyses were observed from microphotographs using an AURIGA CrossBeam scanning electron microscope (SEM, Carl Zeiss Group, Baden-Württemberg, Germany). The surface area and porosity of the samples were estimated by processing the isotherms of the low-temperature adsorption–desorption of nitrogen on a Nova Series 1200e analyzer (Quantachrome Instruments, Boynton Beach, FL, USA) by the Brunauer–Emmett–Teller (BET) and Barrett–Joyner–Halenda (BJH) methods [39,40]. X-Ray diffraction (XRD) spectra of the samples both before and after cycling were obtained using an AXS D8 DISCOVER diffractometer (Bruker) with CuK_α_ source (λ = 1.5418 Å) in the 15–90° range. The average interlayer spacing between aromatic layers (d_002_) was calculated by Bragg’s equation. The size of cobalt crystallites was determined by the Scherrer formula (Equation (1)):

(1) $$D_{Co}=\frac{k\lambda }{\beta cos\theta },$$

where k is the Scherrer constant, λ is the wavelength of X-ray, β and θ are full width at half maximum (FWHM) and the corresponding scattering angle of the diffraction peak, respectively.

Raman spectra were recorded on a Senterra spectrometer (Bruker, Billerica, MA, USA) equipped with a 20-mW 488-nm laser. Fourier-transform infrared (FT-IR) spectra were recorded on a Vertex 70 infrared spectrometer (Bruker) using the Fourier transform in the wave number range of 500–3700 cm^−1^ with a step of 2 cm^−1^. Measurements by X-ray photoelectron spectroscopy (XPS) were carried out on a photoelectron spectrometer Escalab 250Xi (Thermo Fisher Scientific, Waltham, MA, USA) with AlKα radiation (photon energy 1486.6 eV). Spectra were recorded in the constant pass energy mode at 50 eV for element core level spectrum and 100 eV for survey, using an XPS 650 μm spot size with a total energy of 0.2 eV. To counter the surface charge caused by emitting photoelectrons, dual mode charge compensation (a combination of low energy electrons and argon) was used. Investigations were conducted at room temperature in an ultrahigh vacuum of the order of 10^−9^ mbar. Peaks were fitted with a product of asymmetric Gaussian and Lorentzian line shape. Energy-dispersive X-ray (EDX) microanalysis was conducted using an EDX-8100P spectrometer (Shimadzu, Kyoto, Japan). The synchronous thermal analysis of PAN in argon, at a heating rate of 5 °C min^−1^, was performed using a STA 449 F3 Jupiter (Netzsch, Selb, Germany). The analysis combines thermogravimetric analysis (TGA) with differential scanning calorimetry (DSC).

Electrochemical data were recorded using a VMP3 multichannel potentiostat (BioLogic, Seyssinet-Pariset, France) and an Autolab RDE device (Metrohm, Herisau, Switzerland). Measurements were taken in a three-electrode cell using a glassy carbon electrode (GC, S = 0.0707 cm^2^) as the working electrode, a saturated AgCl/KCl electrode as the reference electrode, and Pt wire as the counter-electrode. Electrolyte (0.1 M KOH/H_2_O, pH = 13) was purged with Ar or O_2_ for 10 min prior to the experiment and then kept under the gas flow during measurements. In RDE experiments, continuous O_2_ purge through electrolyte was used in order to maintain the saturated gas concentration.

The catalytic ink was prepared by ultrasonically dispersing 1 mg of carbon-based catalyst in a mixture consisting of 48 µL of deionized water, 89 µL of absolute isopropyl alcohol, and a 71-µL solution of Nafion analogue, perfluorinated sulfonated polymer LF-4SK-I (Plastpolymer, Russia) in isopropanol with a concentration of 7.2 wt.%. The studied materials were applied to the working electrode by the drop-casting of 5 μL of catalytic ink until the catalyst loading on the electrode surface reached 340 μg/cm^2^. For comparison, 20% Pt/C (Vulcan X72) catalyst was used (specific area 150 m^2^/g). The ink with Pt-based catalyst was prepared by the same procedure, the loading was adjusted to correspond to the mass loading of the Co-N catalyst or, in some cases, to provide an equal surface area with the Co-N sample. The electrochemically active surface area of platinum in the Pt/C catalyst was determined by the hydrogen desorption peaks on the cyclic voltammogram in the same alkaline electrolyte used for testing catalytic activity, following the method described in reference [41].

The potential of the working electrode was swept between +0.3 V and −0.9 V vs. AgCl/KCl at 10 mV/s. In the presented data, potentials were converted to the reversible hydrogen electrode (RHE) by adding 0.978 V. The RDE method was used to measure the ORR activity of the investigated materials. The RDE rotation speeds were 500, 1000, 2000, 3000, 4000, and 5000 rpm. Methanol tolerance and long-term stability were tested under the same conditions previously mentioned, the RDE rotation speed was 2000 rmp, and E = 0.678 V vs. RHE. The potential value corresponds to the methanol oxidation peak on Pt-based electrodes in alkaline solutions [42] and matches the ORR activity region of all studied catalysts. The measurement was first carried out for 25 min in the absence of alcohol, then MeOH was added to the electrolyte and the experiment was carried out for a further 25 min.

## 3. Results and Discussion

### 3.1. Catalyst Preparation and Characterisation

Cobalt-doped CN_x_ catalysts were synthesized from PAN in three steps: (i) the impregnation of PAN with a corresponding cobalt precursor by co-precipitation from a DMF solution, (ii) the solvothermal treatment of the Co-impregnated PAN in i-PrOH, and (iii) high-temperature carbonization in an Ar flow. Two types of cobalt precursors were used: Co(OAc)_2_·4H_2_O as a DMF-soluble source of weakly coordinated Co^2+^ ions and the cobalt complex of 1-(2-pyridylazo)-2-naphthol (CoL_2_) as a source of strongly coordinated Co^2+^ ions with the pyridine-containing ligand. The prepared catalysts were labelled according to the cobalt precursor and carbonization temperature (T_carb_) used in their synthesis (Table 1).

**Figure 3:**
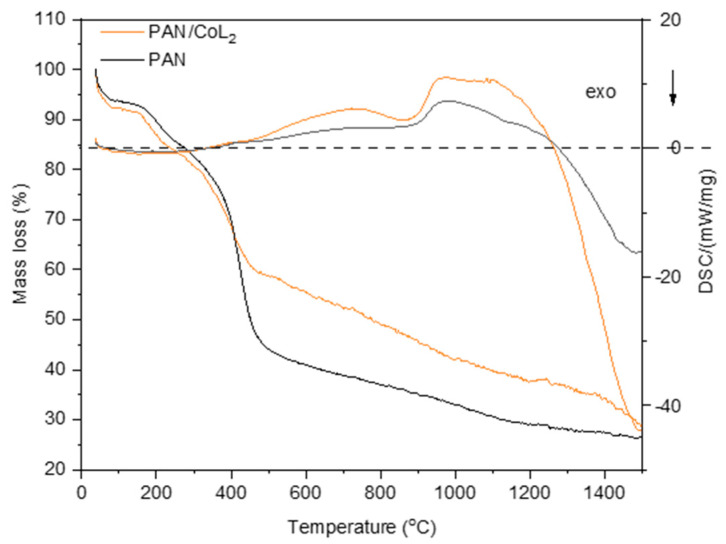
Synchronized thermal analysis heating rate of 5 °C min^−1^.

The thermal degradation mechanism of PAN was investigated by synchronized thermal analysis (Figure 3).

As illustrated in the graph above, the addition of the ligand to PAN does not alter the shape of the mass loss curve. A broad exothermic peak up to 290 °C on the DSC curve, characteristic of both pure PAN and the PAN/CoL_2_ complex, indicates mass loss associated with solvent desorption. The initial stage of carbonization around 400 °C is the result of intra- and intermolecular cyclization and elimination reactions, and is accompanied by an endothermic effect up to 1250 °C [43]. After 1250 °C, a strongly significant exothermic effect for PAN/CoL_2_ is associated with the cyclisation and formation of the graphene structure, accompanied by the loss of nitrogen and the onset of melting of the metal catalyst particles. The presence of a peak around 1480 °C on DSC for PAN and PAN/CoL_2_ indicates the formation of a characteristic thermal effect as a result of the further phase transition of the carbon matrix.

Therefore, the temperatures before and after the phase transition (700 and 1100 °C, respectively) were chosen to evaluate the effect of the carbonization temperature on the activity of the obtained catalysts.

**Figure 4:**
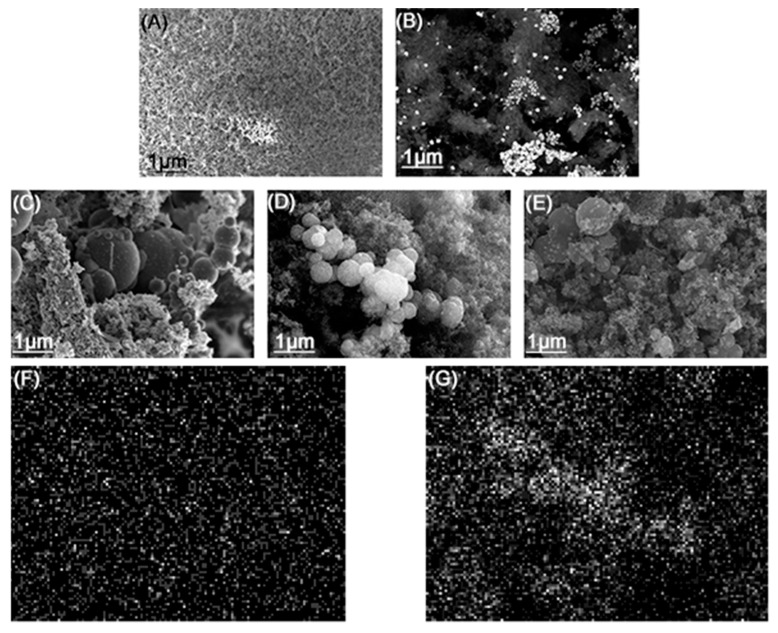
SEM images of the catalysts: (**A**) CN_x_; (**B**) CoAcCN_x_-700; (**C**) CoL_2_CN_x_-700; (**D**) CoL_2_CN_x_-900; (**E**) CoL_2_CN_x_-1100; (**F**,**G**) EDX images of CoL_2_CN_x_-900.

SEM images of the samples (Figure 4A,B) show that CoAcCN_x_-700 and metal-free CN_x_ samples display the typical macroporous–mesoporous morphology, similar to the morphology of Ketjenblack [44]. The surface of CoAcCN_x_-700 is covered with agglomerated particles with an average size of 100–200 nm. The CoL_2_CN_x_ catalyst is characterized by a globular surface morphology with a significant amount of amorphous material. The observed agglomerates in CoL_2_CN_x_ samples exhibit a more uniform surface, which likely facilitates the better dispersion of cobalt centers, contributing to improved catalytic site uniformity compared to the larger metallic particles seen at lower temperatures. The size of particles in CoL_2_CN_x_ samples increased with the carbonization temperature. The sample carbonized at 700 °C contains particles with high contrast on SEM images. These particles have diameters ranging from 20 to 50 nm, and may be attributed to pure Co metal. However, an increase in the calcination temperature leads to the elimination of such high-contrast particles (Figure 4C,D).

On the contrary, larger agglomerates with diameters of 50–100 nm and 100–200 nm are observed for samples obtained at 900 and 1100 °C, respectively. These agglomerates do not show high contrast in the SEM image. To verify the presence of cobalt metal particles not bound to the substrate, an EDX analysis was conducted. In Figure 4F,G, cobalt and carbon mapping for the CoL_2_CN_x_-900 sample, whose SEM image is presented in Figure 4D, are shown. According to the EDX analysis, cobalt atoms are dispersed throughout the material, with no large clusters or metallic particles of nanometer size observed, and the spherical agglomerates are primarily formed by the carbon matrix. A similar pattern is observed for other samples (Figure S1). These results suggest that the cobalt source is an important factor in determining the size, distribution, and morphology of CN_x_ catalysts, while the calcination temperature governs the formation of large Co agglomerates or dispersed Co-C-N material. At the same time, the agglomeration observed at higher temperatures reduces the active surface area, limiting the availability of active sites for ORR and impacting the overall catalytic performance. This highlights a trade-off between improved cobalt dispersion and reduced surface area due to agglomeration.

The evolution of N_2_ gas at decomposition azo-fragment of 1-(2-pyridylazo)-2-naphthol under carbonization conditions enhances the amorphous state of the CoL_2_ surface, and the spherical shaped structures become prevalent in these samples. On the contrary, using Co(OAc)_2_ as a metal source in the PAN matrix makes the carbonized material less homogeneous and leads to the formation of an irregular structure and voids. The heating temperature is the important factor for the appearance of Co in the catalyst. If the sample is pyrolyzed below the phase transition, observed at 800 °C on TGA data, Co forms relatively large metallic particles. The increase in the calcination temperature allows materials with well-dispersed C-N matrix Co atoms to be obtained. The appearance of the materials did not change when the metal source was varied. It was a highly dispersed black powder.

The specific surface area and pore size distribution of the samples were determined by nitrogen adsorption/desorption isotherms measured at liquid nitrogen temperature. The surface area and pore size distribution were calculated with the BET and BJH methods, respectively. Table 2 displays the surface area and micropore volume of the samples prepared at different temperatures.

To validate the reliability of using the BET surface area to approximate the electrochemically active surface area (ECSA), additional calculations based on double-layer capacitance (Cdl) were performed. Cyclic voltammograms were recorded for the samples in an argon atmosphere, and the Cdl values were determined at a potential of 0.5 V. For the ECSA estimation, a specific double-layer capacitance of 20 μF/cm^2^ was assumed for all samples. The results of this analysis are presented in Table 2.

The ECSA values derived from Cdl showed a good correlation with the BET surface area, following similar trends with changes in composition and annealing temperature. The observed differences between the ECSA and BET surface area values can be attributed to the difficulty in precisely determining the specific double-layer capacitance for each material, as well as variations in the effective accessible surface area due to the preparation of catalyst inks involving polymeric binders.

**Figure 5:**
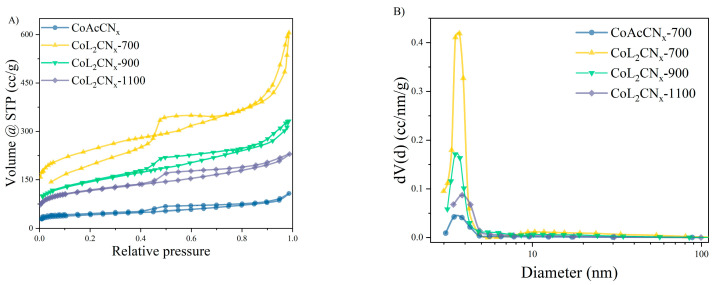
(**A**) Nitrogen adsorption–desorption isotherm; (**B**) pore size distribution curves.

The adsorption/desorption isotherms for all the samples belong to type IV, which is characteristic of mesoporous materials, according to the IUPAC classification (Figure 5A).

The presence of micropores in the samples is indicated by a significant increase in the adsorption line in the 0.05 and 0.1 p/p_0_ region. As evidenced by the intermediate type of hysteresis loops between H_3_ and H_4_, the materials contain both slit and cylindrical pores [45]. The BJH method was used to calculate the structural parameters and pore size distributions, as shown in Figure 5B. The average pore size of all the samples is in the range of 3 to 4 nm. The results highlight the dual function of the CoL_2_ precursor in controlling both the composition and morphology of the resulting material. Using CoL_2_ as the metal source significantly increases the specific surface area compared to the Co(OAc)_2_-derived sample. This provides direct evidence of the amorphization and pore-forming ability of the L fragment.

The specific surface area of the carbon matrix decreases as the carbonization temperature of the CoL_2_-derived CN_x_ sample increases, which is in agreement with the SEM data. However, the values of the specific surface area (S_BET_) and the pore volume (V_pore_) of this sample are still twice as high as those of the sample derived from Co(OAc)_2_.

**Figure 6:**
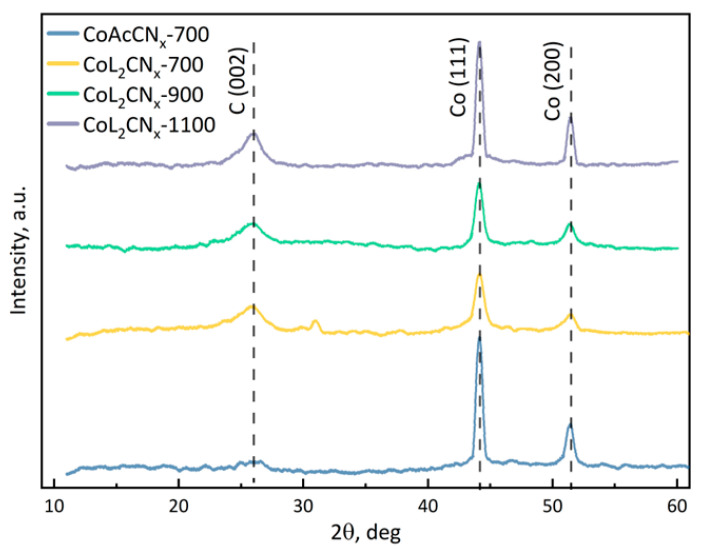
XRD pattern of the catalysts.

The XRD method was employed to analyse the crystalline structure of the carbon and cobalt parts of the catalysts (Figure 6). CoL_2_-derived CN_x_ samples, regardless of the annealing temperature, display a broad peak at 2θ = 26°, corresponding to the graphite (002) planes, which indicates that these samples have highly disordered structures, such as randomly distributed carbon layers. Additionally, there are peaks at 44° and 52°, corresponding to the (111) and (200) planes of metallic cobalt with a face-centred cubic structure. In the X-ray diffraction patterns of samples obtained using CoL_2_, a difference in the peaks of metallic cobalt is observed compared to a Co(OAc)_2_-derived one. For Co(OAc)_2_-derived material, there are pronounced narrow peaks, while CoL_2_-derived CN_x_ demonstrate wide distorted peaks.

Table 3 summarizes the crystal structure parameters of the samples. The average interplanar spacing of the synthesized materials is 0.34 nm. This value is larger than the standard interplanar spacing (0.3354 nm) for pyrolyzed graphite due to the high sp^3^ defect and presence of anionic exfoliated groups between layers [46]. The calculation of the coherent scattering region from the data of the peak corresponding to the plane group (111) of metallic cobalt shows that an increase in the pyrolysis temperature leads to a decrease in the defectiveness of the crystallite. A less defective crystal lattice contributes to the greater stability of the catalyst, and the appearance of defects on the surface of the metal particles as a result of diffusion intensification with increasing temperature provides an enhanced density of active reaction centres.

**Figure 7:**
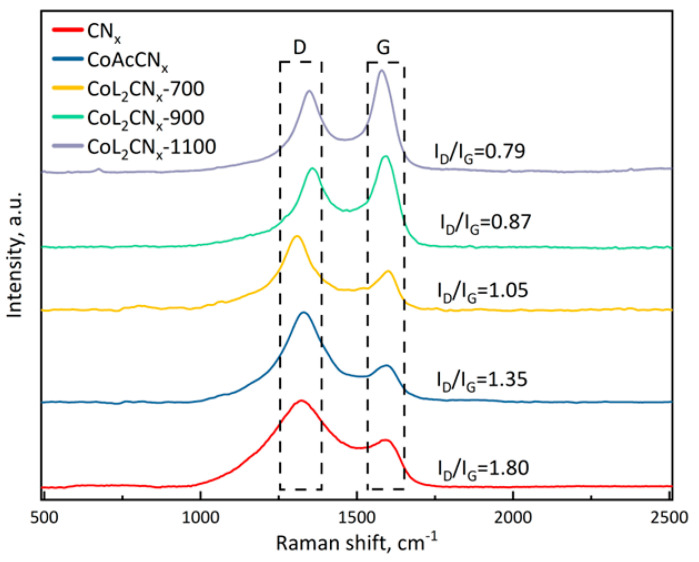
Raman spectra of the catalysts.

Raman spectroscopy of the samples was conducted to refine the results obtained from X-ray phase analysis, specifically to determine the state of carbon atoms in the graphite-like structures. The carbon peak positions of each sample were almost identical at around 1340 and 1600 cm^−1^, corresponding to the D and G bands, respectively. To indicate the degree of the disordered structure of carbon, the ratio of the intensities of these bands, I_D_/I_G_, was used (Figure 7).

The higher ratio indicates a greater prevalence of defects within the structure. The CN_x_ sample had the highest ratio among all the samples. In CoL_2_CN_x_ series, the ratio decreased with an increase in temperature, and was lowest for the CoL_2_CN_x_-1100 sample, in agreement with the XRD results. Furthermore, a shift in the D-band was observed for the CoAcCN_x_-700 sample, while the position of the G-band was maintained. This indicates a highly disordered structure and a significant degree of doping of the carbon structure with nitrogen. Thus, the sample analysis above showed that the carbon structure was influenced by both the pyrolysis temperature and the cobalt source.

**Figure 8:**
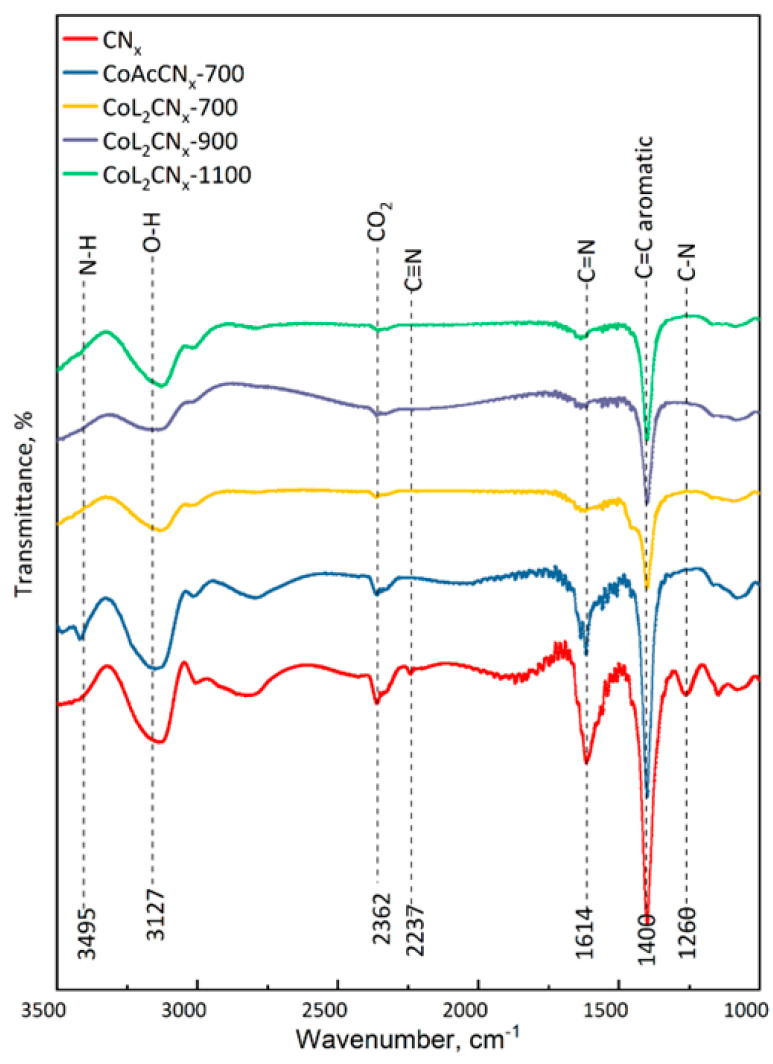
FT-IR spectra of the catalysts.

To confirm the structural changes in the samples, FT-IR was performed to identify characteristic vibrations. The FT-IR spectra (Figure 8) of all samples showed transmission bands due to C-N, C=N, C=C, and C-H vibrations, as well as N-H and nitrile group (C≡N) vibrations.

The bands at 1400 cm^−1^, present in all spectra, correspond to C=C stretching vibrations in the aromatic rings of graphitized carbon. The bands at 1614 and 1260 cm^−1^, most prominent in the CoAcCN_x_-700 and CN_x_ samples, indicate the C=N and C-N vibrations, respectively, corresponding to disordered carbon. The intensities of these bands decrease with the increase in pyrolysis temperature for the CoL_2_CN_x_ samples. The results could confirm structural differences between the samples. The weak band at 2237 cm^−1^, related to the vibrations of the C≡N group, suggests a fundamentally different mechanism of nitrated carbon formation in the metal-doped samples. In addition, the low intensity peak at 2362 cm^−1^ corresponds to atmospheric gaseous CO_2_. The samples also showed bands at 3495 and 3127 cm^−1^ assigned to N-H in compounds and O-H vibrations of water adsorbed on the surface, respectively.

XPS analysis was employed to determine the elemental composition and chemical state of the atoms in the materials. The elemental composition of the samples is summarized in Table 4.

**Figure 9:**
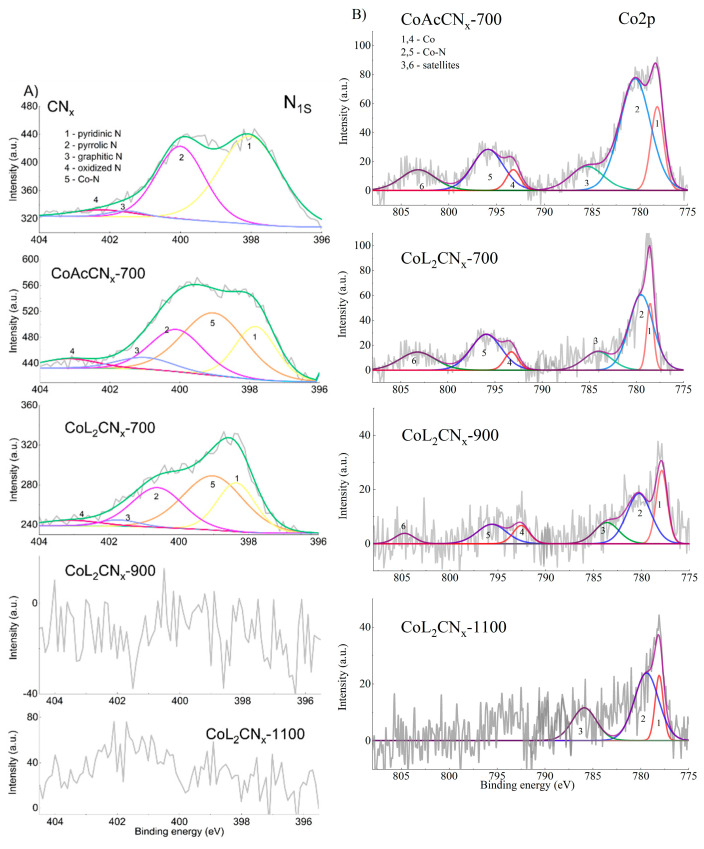
Core-level XPS spectra: (**A**) N_1S_; (**B**) Co_2p_ of the catalysts.

The high-resolution N_1s_ XPS spectrum of CN_x_ was deconvoluted into four nitrogen species, pyridinic N (398.3 eV), pyrrolic N (400.2 eV), graphitic N (401.2 eV), and oxidized N (402.1 eV) (Figure 9A) [47,48]. The nitrogen peaks in the XPS spectra for the CoAcCN_x_-700 and CoL_2_CN_x_ series catalysts have an additional peak at 399.1 eV, which corresponds to the Co-N bond [49]. The appearance of this peak indicates the implementation of cobalt into the structure of the N-doped carbon catalyst during the synthesis process. Pyridinic N and graphitic N were found to be predominant catalytically active sites for anchoring and stabilizing Co atoms. In addition, according to [49], the peak Co-N bond for metal-N-C graphene-based materials was calculated in the range of 399.3–400.9 eV by density functional theory (DFT).

The spectrum in the Co2*p* region was analyzed for the same samples to further confirm the presence of the Co-N bond in the N_1s_ spectrum (Figure 9B). In addition to the peaks due to metallic Co2*p* (~778.2 and 793.3 eV), there are two distinct peaks due to the Co-N bond at ~780.4 and 795.8 eV. This result, as well as EDX, indicates the formation of the Co-N bond in the synthesized Co-N-C catalyst [50,51,52]. The percentage of Co-N goes from about 62 at% at pyrolysis temperature of 700 °C for CoAcCN_x_ and CoL_2_-CN_x_ to 64.5 and 75.6 at% at pyrolysis temperatures of 900 and 1000 °C for CoL_2_ catalysts, respectively (Figure 7). These results further confirm that the Co-N species is the dominant active site for ORR in the synthesized catalysts. The O1*s* spectrum was separated into two peaks at 532.0 and 533.5 eV (Figure S2b), attributed to carbonyl (C=O) and hydroxyl (C-OH) groups, respectively [53,54], and the peak at 530. 2 eV was attributed to Co-O [55,56]. The C1*s* spectrum (Figure S1) was deconvoluted into three peaks at 284.4, 286.2, and 289.1 eV, indicating the presence of C-OH, C=O, imine (C=N), and carboxyl (HO-C=O) [57]. Another peak at 287.7 eV was attributed to the O=C-O bond [57].

As a result, the characterization of the obtained catalyst demonstrates the dependence on the composition and morphology of catalysts from metal precursors. The use of CoL_2_ as a metal source changes the morphology, composition, and specific surface area of the catalysts compared to the Co(OAc)_2_-derived sample. This could prove the amorphization and pore forming ability of L_2_ in CoL_2_-derived catalysts.

### 3.2. Electrocatalytic Properties

The effectiveness of the CoCN_x_ catalysts in the ORR is determined both by the metal source and the calcination temperature.

**Figure 10:**
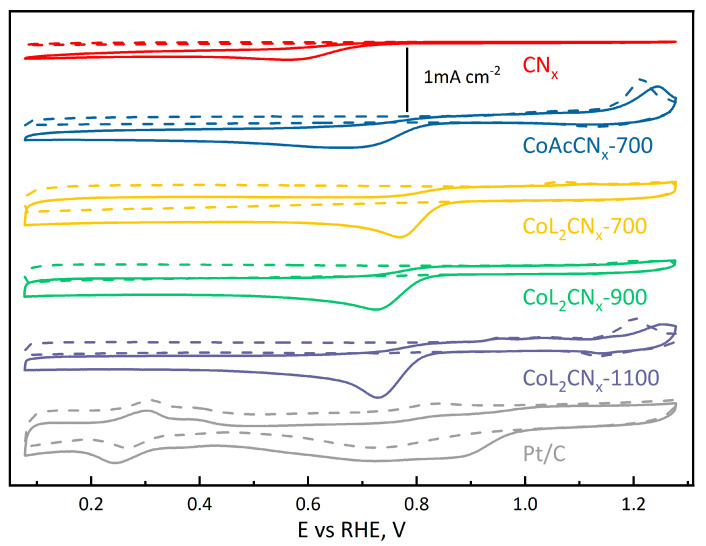
CVs of investigated materials in 0.1 M KOH electrolyte purged with Ar (dashed lines) and O_2_ (solid lines).

To investigate the catalytic activity, cyclic voltammetry (CV) experiments were conducted on each material in Ar-purged and O_2_-saturated electrolytes. In the presence of oxygen, a distinct cathodic peak attributed to the ORR emerges (Figure 10).

**Figure 11:**
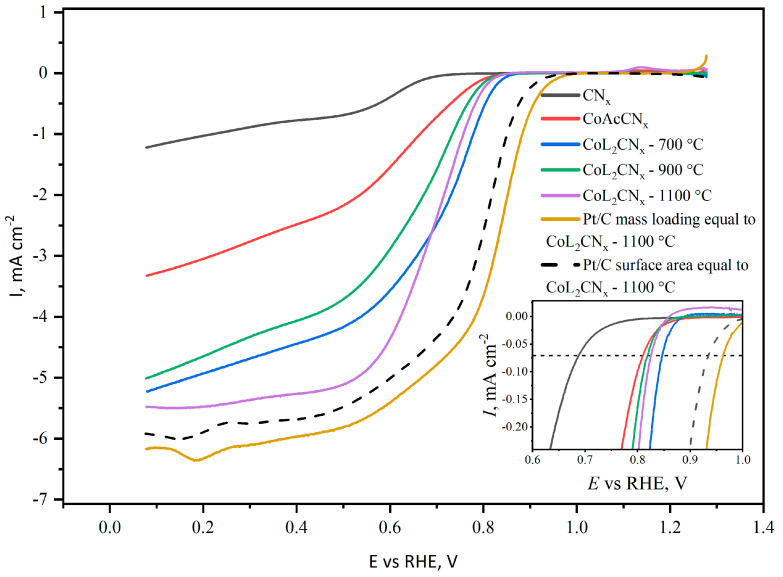
RDE voltammograms of investigated materials in 0.1 M KOH electrolyte purged with O_2_ measured at 5000 rpm (dotted line indicated onset current). Insert is the magnification of onset current region of the CV.

The voltammograms obtained under O_2_-saturated conditions show that all electrode materials discussed exhibit higher ORR overpotentials than the Pt/C electrode, but lower ones than metal-free CN_x_ material. To study the reaction kinetics, the RDE method was used. The corresponding voltammograms recorded at 5000 rpm are shown in Figure 11.

Under these conditions, CN_x_ has the lowest reduction current, which does not reach the diffusion-limited plateau. CoAcCN_x_-700, CoL_2_CN_x_-700, and CoL_2_CN_x_-900 also did not allow the diffusion-limited plateau to be reached due to poor kinetics. Among the materials discussed, CoL_2_CN_x_-1100 is the only one that allows ORR to proceed from the kinetic-limited region to the diffusion-limited region.

To compare the onset potential of ORR on Co-C-N catalysts with Pt as a benchmark material, we used 20% Pt/C catalyst on Vulcan XC72 carbon support. We tested two reference samples, first with equal mass loading, and second with the BET surface area, equal to the CoL_2_CN_x_-1100 sample. In both cases we had to use the BET surface area as the only available characteristics, which describe the Co-based catalyst surface. Despite the possible inhomogeneity in the surface composition, such an estimation may be reasonable for Co-based catalysts calcinated at 900 °C and 1100 °C, since they do not contain large metal agglomerates and therefore may be considered as Co-C-N catalysts. In the case of CoAcCN_x_-700 and CoL_2_CN_x_-700, large metal particles are observed on the surface. This means that the material acts as a mixture of two catalytic materials (N-doped carbon and cobalt metal). Moreover, a strong chemical bond forming between Co and pyridinic N with a high electronegativity leads to the improvement of electron migration from Co to pyridinic N in Co-N catalysts [58]. Then, increasing the concentration of pyridinic N in CoL_2_CN_x_-700 in comparison with CoAcCN_x_-700 (Figure 9) leads to an increase in catalytic activity.

Coupling these catalysts can consist of the generation of H_2_O_2_ by the catalyst with less deep O_2_ reduction, and the further reduction of accumulated H_2_O_2_ on another catalyst. This is reflected on the RDE voltammogram, which has less onset potential than the ORR reaction, but does not reach 4e^-^ diffusion current, even at high overpotential. In this case, the BET value is not a good approximation of a catalytically active surface, as it is not selective, but for Co–carbon composites, the separation of the Co phase area cannot be achieved.

Unlike Co-N samples, the evaluation of the electrochemically active surface area of platinum can be conducted using cyclic voltammetry, focusing on hydrogen desorption peaks, as indicated in reference [41]. According to this evaluation, the electrochemically active surface area of platinum particles is 6 square meters per gram of the Pt/C catalyst (30 m^2^g(Pt)^−1^), while the measured BET surface area of this catalyst is 150 m^2^ × g^−1^. Such a low specific surface area of platinum in the catalyst mass does not allow for the preparation of a catalyst layer containing the same active surface area of platinum particles as in the case of the CoC catalyst, due to the need to apply a large amount of catalyst, which would form thick films with poor adhesion and permeability to the substrate. For this reason, we decided to use only the BET area for the estimation of aerial activity of all the catalysts in the RDE experiments, even in the case of Pt/C, where the electrochemically active Pt surface is certainly less than the BET surface area.

The ORR overpotential was determined as the potential corresponding to the threshold current of 0.05 mA/cm^2^. The obtained values are presented in Table 5. For the Pt/C catalyst, the overpotential values are provided for the same loading as the cobalt–CN_x_ catalyst, either by mass or by specific BET surface area.

The overpotential values for the Co-based materials were found to be around 400 mV, which is 130 mV higher than those of Pt/C. In the case of CoL_2_CN_x_-1100 and Pt/C, a diffusion-limited region was observed, which allowed for the calculation of the corrected kinetic current using Equation (2):

(2) $$i_{k}=\left(i_{l}\times i\right)/\left(i_{l}-i\right)$$

where *i*, *i_l_,* and *i_k_* stand for the current density, diffusion-limited current density, and the kinetic-limited current density, respectively. For the calculation of the kinetic current using Equation (2), the normalization of the currents for the visible area should be done to correct the interpretation of the limiting diffusion current, as the limiting current is proportional to the geometric area. Then the BET surface area may be used as an approximation for the electrochemically active surface area to correct the kinetic current value. Although there are potential errors due to the contribution of currents from metallic cobalt and the carbon nanotube surface, using the BET surface area is justified for the CoL_2_CN_x_-1100 catalyst, as no cobalt agglomerates were observed on the surface, and the catalyst can thus be considered a uniform material. For Pt/C, the electrochemically active surface area was estimated using hydrogen adsorption peaks, which were then applied to calculate the kinetic current, given that the ORR currents on carbon are close to zero in the studied potential range.

**Figure 12:**
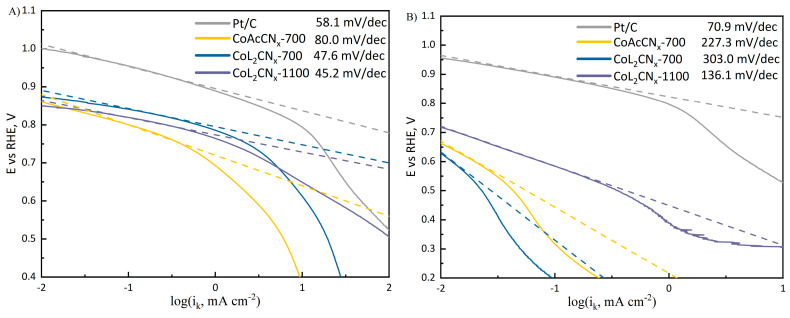
Kinetic currents: (**A**) normalized to the geometric surface area; (**B**) normalized to the real surface area.

Obtained kinetic currents are used to construct Tafel plots. To illustrate the effects arising from different electrochemically active surface areas or the different chemical composition of the catalysts, Tafel plots are shown with kinetic currents normalized using both the geometric (visible) and real surface areas. In Figure 12A, when normalized to the geometric surface area, the Tafel slope for Pt/C was found to be 58.1 mV dec^−1^, which aligns with previously reported values [59], while the slope for CoL_2_CNx-1100 was 45.2 mV dec^−1^, indicating superior activity. However, CoAcCN_x_-700 showed the poorest performance, with a slope of 80 mV/dec.

When normalized to the real surface area, as shown in Figure 12B, the Tafel slopes for both CoL_2_CN_x_ and CoAcCN_x_-700 converged, not exceeding 200 mV/dec. This suggests that, at the annealing temperature used for CoAcCN_x_-700, the ligand from which the catalyst was derived did not enhance the catalytic activity of the active centers. The overall higher activity of CoL_2_CN_x_ compared to CoAcCN_x_ is attributed to increased porosity and a greater available surface area due to nitrogen release during the pyrolysis of CoL_2_CN_x_. Meanwhile, for CoL_2_CN_x_-1100, the Tafel slope was 136 mV dec^−1^, approaching the value for Pt/C (80 mV dec^−1^), indicating that at higher temperatures, the material gains additional activity.

In conclusion, at lower annealing temperatures, the ligand in CoL_2_CN_x_ primarily enhances activity due to pore formation, whereas at higher temperatures the material acquires additional catalytic activity due to the formation of active Co-C-N centers.

It is notable that the developed catalysts exhibit comparable activity to catalysts reported in other publications (Table 5). The utilization of the template-synthesis method, including the employment of zinc compounds as pore-forming agents, resulted in the formation of the Co-N-C catalyst structure, which exhibited comparable outcomes to those observed in this study for the initial reaction potential. According to the results of the determination of the physico-chemical properties of the obtained materials, the systematically higher specific surface area and the total pore volume with the preservation of the predominant microporosity, as well as the higher content of nitrogen atoms, does not significantly affect the catalytic activity of Co-N-C in the ORR. The data obtained and presented by us prove that the availability of active centers, including both the size of the metal particles themselves and the pore diameter in the carbon matrix, plays a crucial role in catalysis. The latter implies the difficulty of oxygen transport to the active sites.

**Figure 13:**
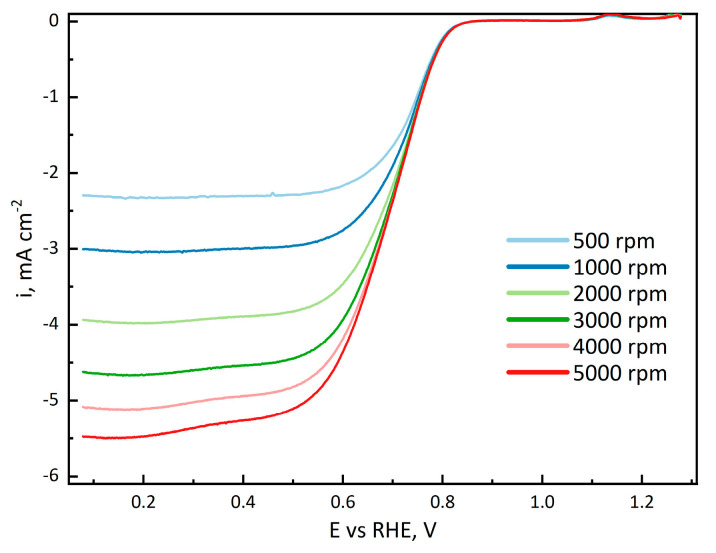
RDE cyclic voltammetry of CoL_2_CN_x_-1100 in 0.1 M KOH electrolyte purged with O_2_.

The ORR currents at different rotation rates for the CoL_2_CN_x_-1100 catalyst are also illustrated in Figure 13.

**Figure 14:**
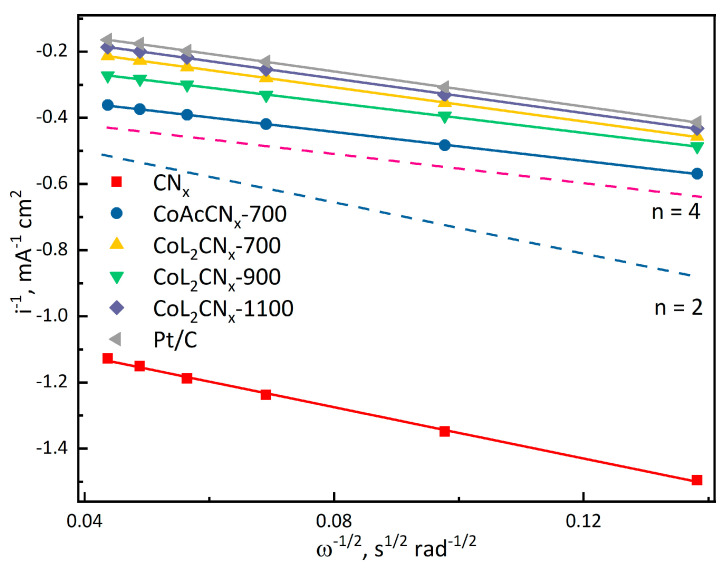
Levich–Koutecky analysis of RDE CVs of investigated materials in 0.1 M KOH electrolyte purged with O_2_.

The difference in the number of electrons involved in the ORR is responsible for the difference in the diffusion currents between CoL_2_CN_x_-1100 and Pt/C. The electron number was estimated by the Levich–Koutecky (LK) method (Figure 14). The LK plots for E = 0.3 V vs. RHE show linear dependencies for both the Co-based and Pt/C catalysts, with slopes approaching the 4-electron mechanism of the ORR, with the exact electron number for CoL_2_CN_x_-1100 being 3.8.

Figure 14 presents the dependencies normalized to the apparent surface area. This is necessary for the correct accounting of diffusion currents. The extrapolation of the dependency in Levich–Koutecky coordinates allows for the determination of the kinetic currents that define the surface activity of the catalyst. In this case, for the Pt/C catalyst, it is necessary to recalculate the current, normalizing it to the electrochemically accessible surface area, which will account for the activity of the platinum particles specifically. Under these conditions, the kinetic current on platinum reaches 2 mA cm^−2^, while on the best cobalt catalyst sample it is 0.05 mA cm^−2^, and on the CN_X_ catalyst produced without cobalt it is only 0.005 mA cm^−2^. Moreover, the catalyst obtained from cobalt acetate (CoAcN-CN_X_700) exhibits catalytic activity that is twice as low as that of CoL_2_CN_x_-700. In this case, the kinetic current is 0.03 mA/m^2^.

To provide additional evaluation of the catalyst’s performance, a comparative analysis was conducted between the catalyst studied in this work and other similar PAN-Co-N-doped electrocatalysts reported in the literature. The results, summarized in Table 6, highlight the catalytic activity parameters, including onset potential and limiting current density.

**Figure 15:**
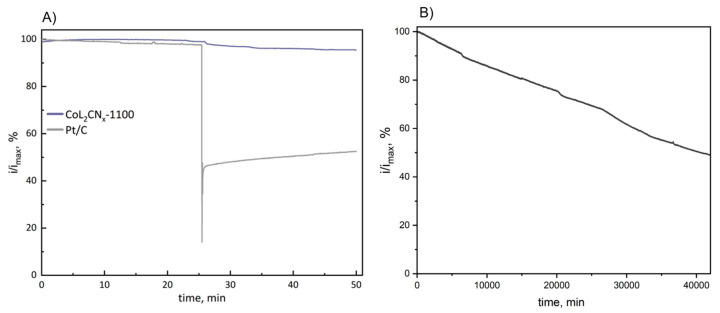
(**A**) Comparative methanol tolerance of CoL_2_CN_x_-1100 C and Pt/C. For each material, the current is normalized to its maximum value. (**B**) Long-term stability test of CoL_2_CN_x_-1100 °C.

The most efficient CoL_2_CNx-1100 catalyst was tested for its tolerance to methanol under the conditions of an O2-saturated electrolyte, 2000 rpm electrode rotation rate, and E = 0.678 V vs. RHE. The current decay before and after addition of 0.1 M MeOH is depicted in Figure 15A, which clearly indicates that CoL_2_CN_x_-1100 showed almost no reaction to the presence of methanol, while Pt/C reacted with a sharp decrease in current. In summary, CoL_2_CN_x_-1100 is the most efficient catalyst among the tested materials.

The long-term stability of the CoL_2_CN_x_-1100 catalyst was evaluated at E = 0.678 V vs. RHE over 40,000 min under constant operating conditions. The results, presented in Figure 15B, demonstrate that the catalyst retains approximately 50% of its initial activity after this extended period, showcasing its stability for prolonged use. These results underscore the CoL_2_CN_x_-1100 catalysts as stable and durable materials, making them promising candidates for ORR applications in practical environments.

## 4. Conclusions

In this study, we implemented the strategy for the synthesis of Co, N-codoped carbon catalysts using a PAN matrix and a cobalt complex with a well-known acid-base indicator and spectrophotometric reagent, 1-(2-pyridylazo)-2-naphthol, as the metal source. We demonstrated that the nature of the metal source and carbonization temperature strongly affect the morphology of the obtained CN_x_ materials. The characterization of the materials by means of SEM, BET, XRD, FTIR, XPS, and Raman spectroscopy methods indicated that the 1-(2-pyridylazo)-2-naphthol-derived catalysts exhibited a substantially higher surface area, total pore volume, smaller Co grain size, and higher pyridinic N content compared with acetate-derived samples obtained at the same temperature, which are characterized by more disordered graphitic planes and the higher fraction of sp^3^ carbons. The observed difference is reflected in a reduction in overpotential, and an increase in ORR current and electron transfer number for the CoL_2_-derived catalyst. The obtained results confirm the role of the CoL_2_ as a pyridinic N source, metal dispersant, and blowing agent.

The carbonization temperature of the CoL_2_-impregnated PAN also affects the structure and morphology of the resulting materials. As temperatures increase, the CoL_2_CN_x_ materials become increasingly ordered, exhibiting a lower surface area and pore volume. The electrochemical test results indicate that, while increased porosity enhances the catalytic activity of CoL_2_CN_x_ compared to the acetate-based catalyst at lower carbonization temperatures, higher temperatures lead to structural changes that grant the material additional catalytic activity. Despite the reduction in surface area and porosity, the CoL_2_CN_x_-1100 catalyst demonstrates the limiting diffusion current with a plateau at 5.5 mA cm^−2^ and the highest electron transfer number. Furthermore, the overpotential remains nearly constant across all samples within the calcination temperature variation series.

In conclusion, the development of an affordable catalyst, prepared from inexpensive and abundant materials via a straightforward method, presents a promising opportunity for advancing and producing a commercial Pt-free ORR catalyst. Despite the moderate activity of the studied catalyst, the combination of pore-forming and nitrogen-doping fragments in the CoL_2_-source used for the preparation of the catalysts holds potential for the further development of materials for practical applications.

## Figures and Tables

**Table 1:** Labels, Co precursors, and carbonization temperatures of prepared catalysts.

Label	Co Precursor	T_carb_, °C
CN_x_	-	700
CoAcCN_x_-700	Co(OAc)_2_·4H_2_O	700
CoL_2_CN_x_-700	CoL_2_	700
CoL_2_CN_x_-900	CoL_2_	900
CoL_2_CN_x_-1100	CoL_2_	1100

**Table 2:** Physical structure parameters of samples.

Sample	CoAcCN_x_-700	CoL_2_CN_x_-700	CoL_2_CN_x_-900	CoL_2_CN_x_-1100
Surface area (BET/double layer capacity), m^2^ × g^−1^	71.6/80	440.3/250	210.3/150	150.2/120
S_micro_, m^2^ × g^−1^	44.1	231.8	112.3	86.4
V_pore_, cc × g^−1^	0.12	0.79	0.34	0.22
Average pore diameter, nm	3.45	3.71	3.50	3.85
Micropore percentage, %	61.6	52.6	53.4	57.5

**Table 3:** Crystal structure parameters of samples derived from XRD pattern.

Sample	Bragg Angle (°)	d_002_ (nm)	2θ (°)	β (Radian × 10^−2^)	D_Co_ (nm)
CoAcCN_x_-700	25.79	0.345	44.46	3.09	22.4
CoL_2_CN_x_-700	25.95	0.344	44.10	4.77	18.8
CoL_2_CN_x_-900	25.94	0.343	44.12	4.17	23.0
CoL_2_CN_x_-1100	25.87	0.345	44.22	4.25	24.2

**Table 4:** Composition of surface of the catalysts.

Sample	CoAcCN_x_-700	CoL_2_CN_x_-700	CoL_2_CN_x_-900	CoL_2_CN_x_-1100
T_carb_	700	700	900	1100
C, at.%	84.0	84.3	93.3	95.44
N, at.%	4.5	2.92	1.28	0.63
O, at.%	9.9	12.75	4.94	3.7
Co, at.%	1.5	0.44	0.44	0.26

**Table 5:** Onset current potentials and calculated ORR overpotentials.

Material	CN_x_	CoAcCN_x_-700	CoL_2_CN_x_-700	CoL_2_CN_x_-900	CoL_2_CN_x_-1100	Pt/C
E, V	0.69	0.82	0.85	0.82	0.83	0.96
1/i_k_, cm^2^/mA	196.9	39.1	90.9	73.9	21.9	0.5
η, mV	542	407	383	412	404	267 (295)

**Table 6:** Comparison of catalytic activity parameters for PAN-Co-N-Doped catalysts in ORR.

Sample	Precursor	T_pyrolysis_, (K)	E_onset_, (V vs. RHE), 0.1 M KOH	Tafel Plot Slope (mV × dec^−1^)	Ref.
CoL_2_CNx-1100	Co complex of 1-(2-pyridylazo)-2-naphthol)	1100	0.83	45.0	This work
Co–N-CNFs-0.2-800	Co/Zn PAN@BZIFs	800	0.96	60.2	[60]
FeCo/N-C CNFs	Zn(Ac)_2_·2H_2_O, Co(Ac)_2_·4H_2_O, FeCl_3_·6H_2_O	800	0.99	80.0	[28]
MN_4_-PAN-A	Fe(II)phthalocyanine, Co(II)phthalocyanine	900	0.90	71.2	[31]
Co–SiCN(O)-700 *	Co(NO_3_)_2_·6H_2_O	700	0.83	-	[61]
Co-PAN-A1000	Co(OAc)_2_	1000	0.91	-	[62]
Co-N-C@PAN (PAN@ZIF-67)	CoCl_2_	800	1.05	72.4	[29]
Co-N-C (ZIF-67 NPs)	CoCl_2_	800	0.91	80.8	[29]
NiFe@C@Co CNFs	Ni(OAc)_2_,Fe(acac)_3_, Zn(Ac)_2_, Co(NO_3_)_2_·6H_2_O	800	0.94	57.0	[63]
CoN-CS-800	Zn(NO_3_)_2_·6H_2_O, Co(NO_3_)_3_·9H_2_O	800	0.88	93.0	[30]
FeCo@MNC	Co(NO_3_)_2_·6H_2_O, FeCl_3_·6H_2_O	900	-	66.0	[32]
Co−N−C(A)	Co-ZIF-8	900	1.01	59	[27]
Co−N_3_C_1_@GC	Co/Zn-ZIF@ZIF-6	900	0.91	46	[64]
Co_1_−N_3_PS/HC	Co-ZIF-8@PZS	950	1.00	31	[65]
Co-pyridinic N−C	Co-ZIF-8@Lys	1100	0.99	98	[26]
RuCo@Co−N−C	RuCo-ZIFs	700	-	51	[66]

* Experiments in 1 M KOH solution.

## Data Availability

Data are contained within the article and Supplementary Material.

## Supplementary Materials

The following supporting information can be downloaded at: https://www.mdpi.com/article/10.3390/nano14231924/s1. Figure S1 EDX data for CoL_2_CN_x_ – 1100 catalyst; Figure S2 XPS spectra of the catalysts on atoms (a) O_1s_ (b) C_1s_; Figure S3. CV of Pt-C coated electrode in 0.1 M KOH electrolyte purged with O_2_.
