# Supplementary material for: Impact of Metal Source Structure on the Electrocatalytic Properties of Polyacrylonitrile-Derived Co-N-Doped Oxygen Reduction Reaction Catalysts
Source: Nanomaterials (Basel). 2024 Nov 29;14(23):1924. doi: 10.3390/nano14231924 (PMC11643432; doi:10.3390/nano14231924)
Supplement: Supplementary file 1 [file nanomaterials-14-01924-s001.zip › nanomaterials-3313126-supplementary.pdf]

Supplementary materials

Impact of Metal Source Structure on the Electrocatalytic Properties of Polyacrylonitrile-Derived Co-N-Doped Oxygen Reduction Reaction Catalysts

Arseniy Kalnin 1 , Ksenia Kharisova 1 , Daniil Lukyanov 1 , Sofia Filippova 1 , Ruopeng Li 2 , Peixia Yang 2 , Oleg Levin 1 and Elena Alekseeva 1, *

1 Electrochemistry Department, St. Petersburg State University, 7/9 Universitetskaya nab., 199034 St. Petersburg, Russia

2 State Key Laboratory of Urban Water Resource and Environment, School of Chemistry and Chemical Engineering, Harbin Institute of Technology, Harbin 150001, China

* Correspondence: e.v.alekseeva@spbu.ru; Tel.: +7-812-428-69-00

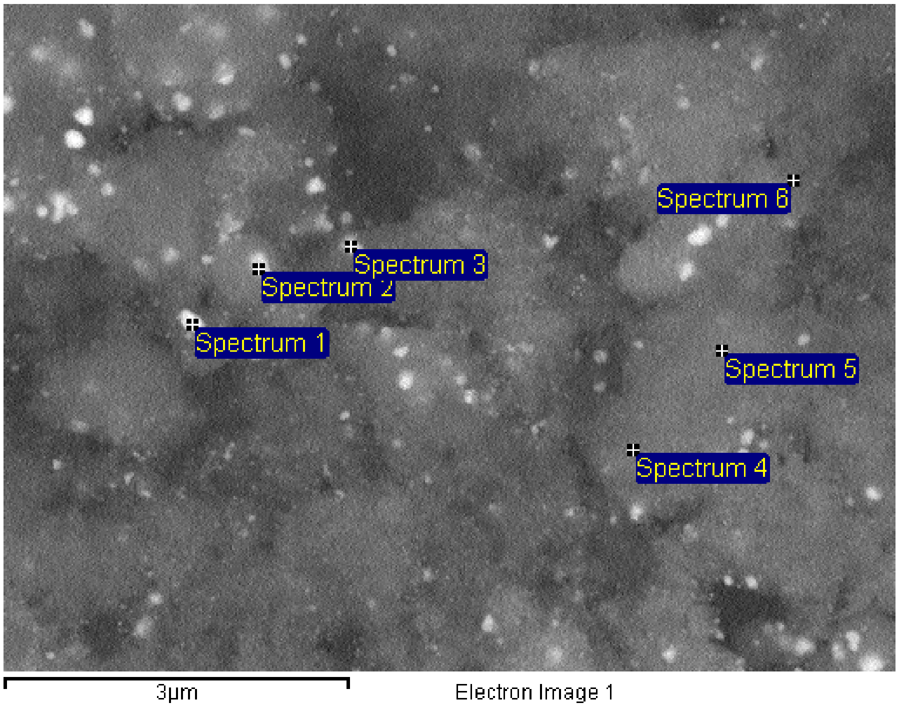

Spectrum	In stats.	C	O	Co
Spectrum 1	Yes	83.23	0.00	16.77
Spectrum 2	Yes	86.78	1.26	11.96
Spectrum 3	Yes	89.83	0.79	9.39
Spectrum 4	Yes	94.60	1.60	3.80
Spectrum 5	Yes	94.12	1.43	4.45
Spectrum 6	Yes	94.64	2.29	3.06
Mean		90.53	1.23	8.24
Std. deviation		4.78	0.78	5.46
Max.		94.64	2.29	16.77
Min.		83.23	0.00	3.06

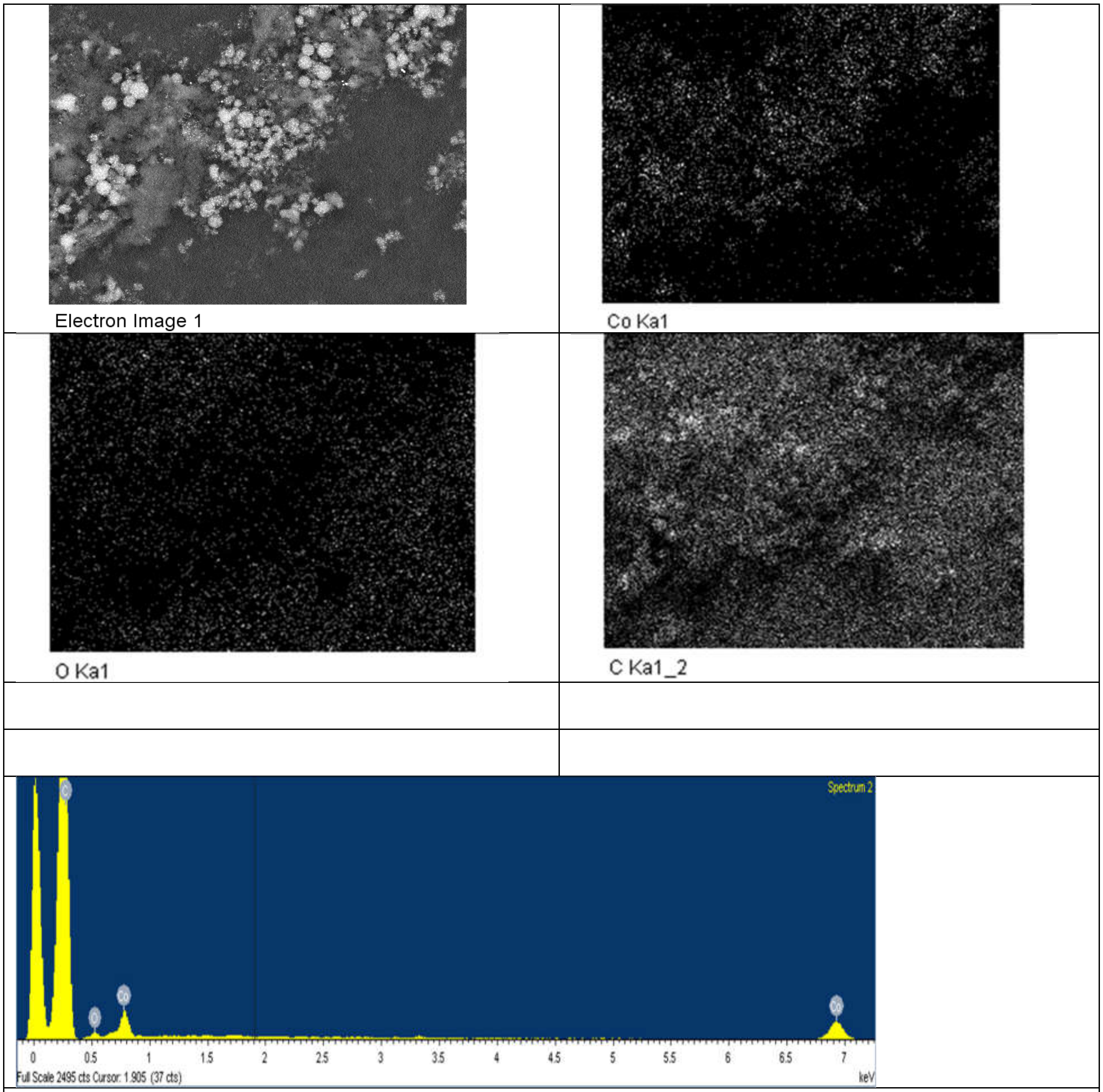

Sample	CN_x	CoL_2CN_x-700	CoL_2CN_x-900	CoL_2CN_x-1100
T_{carb}	700	700	900	1100
C, at.%	78.55	84.79	92.31	95.53
N, at.%	26.69			
O, at.%	3.35	7.14	2.71	2.52
Co, at.%	0	3.73	1.04	1.95

Figure S1 EDX data for CoL_2CN_x – 1100 catalyst As presented in Fig. S1 there are no cobalt agglomerates in the sample.

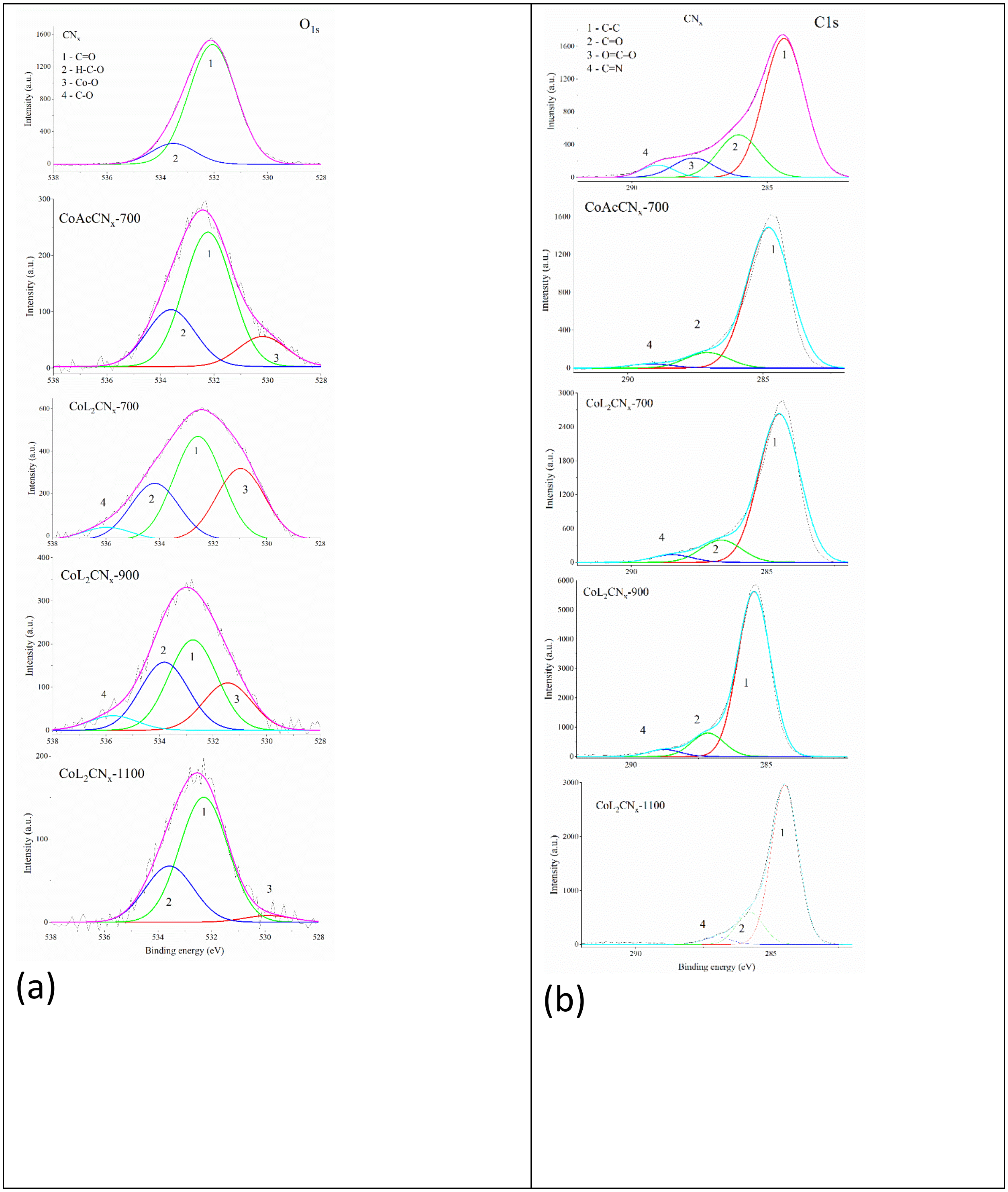

Figure S2. XPS spectra of the catalysts on atoms (a) O_{1s} (b) C_{1s}.

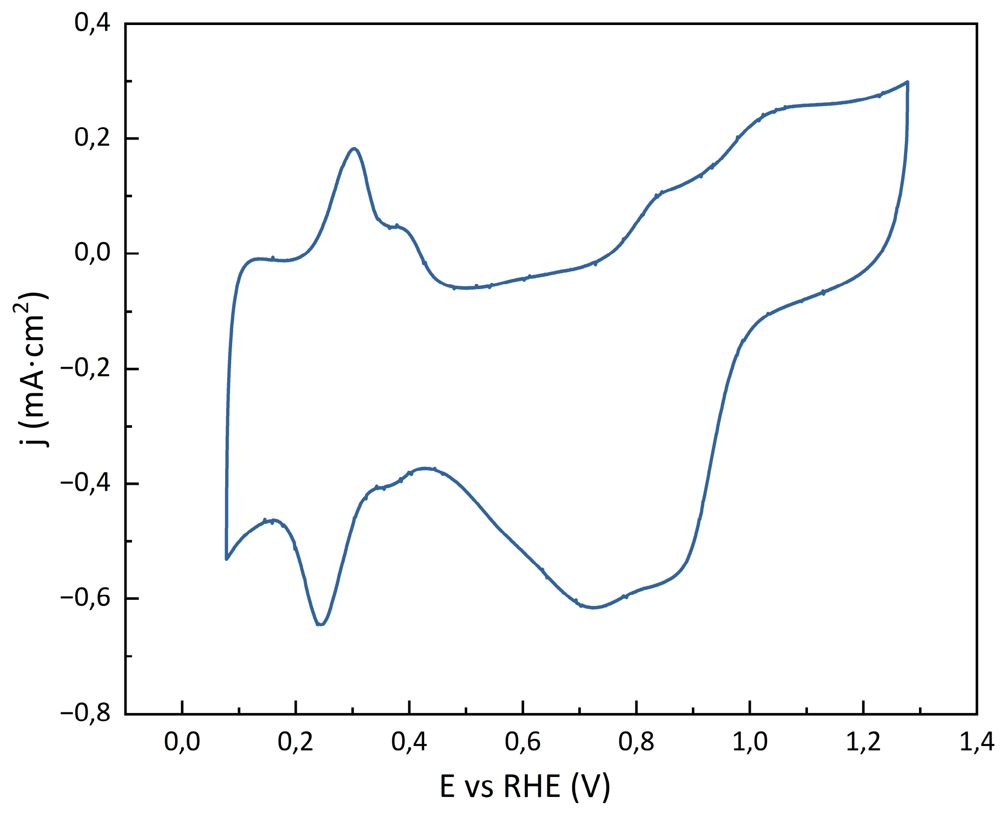

Figure S3. CV of Pt-C coated electrode in 0.1M KOH electrolyte purged with O_2.
